# Of Money and Trust in Biomedical Care

**DOI:** 10.4103/0973-1229.32143

**Published:** 2007

**Authors:** Joel Lexchin

**Affiliations:** School of Health Policy and Management, Faculty of Health, York University, 4700 Keele St, Toronto Ontario M3J 1P3, Canada E-mail: jlexchin@yorku.ca

Over the past few years we have been repeatedly told that we now live in an evidence-based medical world. That should be good news since evidence comes from science. But on a deeper level doctors’ and the public's faith in science rests on trust; trust that the right questions have been asked, trust that the clinical trials have been done correctly, trust that the results of the trials have been interpreted in the proper manner and trust that what finally appears in medical journals and clinical practice guidelines accurately reflects what is known.

## Erosion of Trust

However, this trust is being eroded because of the growing links between the pharmaceutical industry and medicine. At the start of 2006 an influential group of American academics proposed the complete elimination of practices that lead to situations of potential conflict-of-interest in academic medical centres (Brennan *et al.*, 2006).

A few years ago few people would have advocated such a dramatic move but accumulating research has shown the dangers of maintaining such a liaison. Gifts, in the form of trips to resort areas to hear about the virtues of new drugs, can dramatically affect the prescribing behaviour of doctors even when the new drug has no advantage over existing ones and the doctors themselves, before they left on the trip, denied that they could be swayed by such favours (Orlowski *et al.*, 1992). Denial that they can be influenced is a common finding amongst doctors. Only 1% of residents admitted that they could be influenced a great deal by pharmaceutical sales representatives but they were less certain about their colleagues. They felt that a third of their colleagues could be influenced a great deal and another 16% could be influenced a bit (Steinman *et al.*, 2001).

A survey of academic scientists in the United States in the mid 1990s found that over one-third with industry funding took commercial considerations into account when choosing research topics compared to less than 15% for those without such sponsorship (Blumenthal *et al*., 1996a). If choice is determined by the source of money then many areas of medicine will be bereft of research and evidence. Not only is the choice of topic affected by where the money comes from but so too is the willingness of people to share the results of their research, either in the form of information or biomaterials (Blumenthal *et al., 1996a*; *Blumenthal et al., 1996b*).

Academic institutions are willing to let industry retain control over crucial aspects of research in return for the money that their faculty receives. Although 85% of 107 institutions that were surveyed would not approve provisions giving industry sponsors the authority to revise manuscripts or decide whether results should be published, 50% allowed industry personnel to draft manuscripts and 62% would agree to keep the terms of the clinical-trial agreement confidential (Mello *et al.*, 2005).

Industry funding for continuing medical education (CME) has been steadily increasing compared to revenue from registration fees and other income and now accounts for about 50% of the money that goes into this area (Rutledge *et al.*, 2003). CME financed by industry results in a narrower range of topics being presented and, given the economic interests of the drug companies, these topics are generally ones that primarily have a pharmaceutical solution (Katz *et al.*, 2002). Once again doctors are quick to deny that there are any downsides to attending an event paid for by industry (Rutledge *et al.*, 2003).

In order to help sales of Xigris (recombinant human activated protein C) in the treatment of sepsis Eli Lilly financed the development of a three-pronged marketing strategy that resulted in the publication of guidelines for sepsis management in 2004. Missing from subsequent editions of these guidelines was any mention of the magnitude of the increase in serious risk that can result from use of Xigris (Eichacker *et al.*, 2006).

Even the results of meta-analyses are subject to bias when industry is involved. Seven industry supported meta-analyses that compared two drugs recommended the drug from the sponsor without reservation whereas none of the meta-analyses sponsored by the Cochrane Collaboration made such a recommendation. Industry supported reviews were less transparent and had fewer reservations about the methodological limitations of the included trials (Jørgensen *et al.*, 2006).

### Occasional benefits but greater harm

Is it possible for contacts between academic doctors and industry to have beneficial effects in the public interest as opposed to the individual and corporate interests of the doctors and drug companies, respectively? In almost all areas, the evidence cited above suggests that while there may be occasional benefits the risk of harm is much greater. Therefore, I would argue that doctors should not accept gifts of any sort. Likewise free samples should be refused and replaced by a system of vouchers to allow low income patients to receive medications without charge (Brennan *et al.*, 2006). Doctors need to divorce themselves from their reliance on the pharmaceutical industry to fund their CME. No other profession receives such largesse from a commercial entity to pay for what is an essential element of being a professional. And in addition, doctors are near the top of the professional pay scale and can afford to pay for their own CME.

However, it is also not realistic to suppose that there will never be interactions between the two parties. One area where engagements may be acceptable is consulting for industry. However even here there needs to be strict enforcement of boundaries. As Brennan and colleagues advocate:

*… consulting or honoraria for speaking should always take place with an explicit contract with specific deliverables and the deliverables should be restricted to scientific issues… A contract with no identified deliverables is tantamount to a gift and should be regarded as such (*Brennan *et al*., 2006*)*.

Furthermore, all such arrangements along with the amounts of money involved and names of the parties should be posted on a publicly available web site and this site should be up-dated on a regular basis.

The biases that come with the money from pharmaceutical companies are rapidly eroding the trust that doctors and the public have in medical science. Once trust has been lost mere attestations of good faith will not be enough to restore it. We can no longer be content to just issue bland statements to the effect that the researcher or author has no conflicts-of-interest or to list the companies that we have a relationship with. Now is the time for bold aggressive action to retain trust before it is too late.

## About the Author

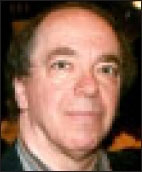



Dr. Joel Lexchin received his MD from the University of Toronto and works as an emergency physician. He is a Professor in the School of Health Policy and Management at York University and was a consultant for the province of Ontario, various arms of the Canadian federal government, the World Health Organization and the government of New Zealand. He is the author or co-author of over 70 peer-reviewed articles in the area of pharmaceutical policy. He is a member of Healthy Skepticism Inc. an Australian based organization that works to improve health by reducing harm from misleading drug promotion.
